# Adapting Highly-Dynamic Compliant Movements to Changing Environments: A Benchmark Comparison of Reflex- vs. CPG-Based Control Strategies

**DOI:** 10.3389/fnbot.2021.762431

**Published:** 2021-12-10

**Authors:** Annika Schmidt, Benedikt Feldotto, Thomas Gumpert, Daniel Seidel, Alin Albu-Schäffer, Philipp Stratmann

**Affiliations:** ^1^Sensor Based Robotic Systems and Intelligent Assistance Systems, Department of Informatics, Technical University of Munich, Garching, Germany; ^2^German Aerospace Center (DLR), Institute of Robotics and Mechatronics, Weßling, Germany; ^3^Robotics, Artificial Intelligence and Real-Time Systems, Department of Informatics, Technical University of Munich, Garching, Germany

**Keywords:** robotics, stability, energy efficiency, bioinspired control, feedback, performance metric

## Abstract

To control highly-dynamic compliant motions such as running or hopping, vertebrates rely on reflexes and Central Pattern Generators (CPGs) as core strategies. However, decoding how much each strategy contributes to the control and how they are adjusted under different conditions is still a major challenge. To help solve this question, the present paper provides a comprehensive comparison of reflexes, CPGs and a commonly used combination of the two applied to a biomimetic robot. It leverages recent findings indicating that in mammals both control principles act within a low-dimensional control submanifold. This substantially reduces the search space of parameters and enables the quantifiable comparison of the different control strategies. The chosen metrics are motion stability and energy efficiency, both key aspects for the evolution of the central nervous system. We find that neither for stability nor energy efficiency it is favorable to apply the state-of-the-art approach of a continuously feedback-adapted CPG. In both aspects, a pure reflex is more effective, but the pure CPG allows easy signal alteration when needed. Additionally, the hardware experiments clearly show that the shape of a control signal has a strong influence on energy efficiency, while previous research usually only focused on frequency alignment. Both findings suggest that currently used methods to combine the advantages of reflexes and CPGs can be improved. In future research, possible combinations of the control strategies should be reconsidered, specifically including the modulation of the control signal's shape. For this endeavor, the presented setup provides a valuable benchmark framework to enable the quantitative comparison of different bioinspired control principles.

## 1. Introduction

It is one of the longest standing goals of neuroscience to decode how the mammalian central nervous system (CNS) controls locomotion. Until the beginning of the twentieth century, it was widely assumed that such locomotion was purely triggered and controlled by sensory feedback in the form of *reflexes*. Brown (1911) questioned this assumption and proposed instead the presence of what is now known as *Central Pattern Generators (CPGs)*: coordinated patterns of periodic activity that emerge without periodic input from sensory feedback from higher control centers (Ijspeert, 2008). With time, experiments have supported the notion that both control mechanisms, reflexes and CPGs, are important to control highly-dynamic compliant movements in all limbs (Ivanenko et al., 2006), even when they are not primarily used for locomotion (Zehr and Chua, 2000). Thereby sensory information is not only essential for the reflex control, but is also necessary to adapt the oscillations of CPGs to body motion, especially during unexpected disturbances (Mellen et al., 1995; Rossignol et al., 2000; Raibert et al., 2008). However, the degree to which reflexes and CPGs influence the control of highly-dynamic compliant movements is still a fundamental open question in neurosience.

In the ongoing quest to understand biological control mechanisms, we can seek inspiration from modern control approaches that have been engineered for robots that mimic the dynamics of the mammalian locomotor system. Robotic control theory comprises a range of well-tested analytical frameworks that can help to analyze bioplausible control approaches. This can provide useful insights into how the brain implements different control principles (Pearson et al., 2006; Jagacinski and Flach, 2018; Stratmann et al., 2018). In simplified technological terms, a pure reflex controller can be regarded as a static state feedback controller, while pure CPG control translates to a time-based feedforward strategy. In this form, both control approaches have been longstanding subjects to robotic investigations of limit cycle motions, mainly applied to quadrupedal (Tsujita et al., 2001; Ferreira et al., 2015) and bipedal locomotion (Endo et al., 2004; Liu et al., 2019). However, only more recently have these approaches been applied to biomimetic models that take into account the major constraints of the CNS.

In contrast to static walking, where a system is always supported by a stable base of at least three legs, highly-dynamic gaits, such as running or hopping, rely on continuous movements to achieve stable motions. The leg is not only regarded as an inverted pendulum, but additionally incorporates an elastic element, i.e., a spring, which allows the system to take advantage of intrinsic mechanical dynamics for stability and robustness (Maus et al., 2010). As multiple studies based on such spring-loaded inverted pendulums have shown (Schwab and Wisse, 2001; Seyfarth et al., 2003), passive dynamic walkers can withstand small disturbances even without the need of any control when properly designed. But in order to recreate the mammalian flexibility to adjust their motions or to react to disturbances, control mechanisms are necessary. A reflex as pure feedback control is the simplest control extension and can achieve relatively stable and adaptive locomotion in bipedal robots (Manoonpong et al., 2007). Taking into account the constraints of the CNS, Geyer and Herr (2010) proposed a more realistic neuro-musculoskeletal model for a compliant system that was driven solely by bioplausible reflex loops. In simulations, the system was able to resist disturbances without a CPG component. However, the research also showed that when relying only on feedback control deliberate gait modifications are challenging, i.e., modulating the gait by changing speed or step length, thus limiting the flexibility of pure reflex control. Such gait modulations become easier when the CPG component is added to the proposed neuro-musculoskeletal model as proposed by Dzeladini et al. (2014) and applied to a bipedal model by Greiner et al. (2018). While the application of this control strategy leads to promising results in theory (Greiner et al., 2018), it also becomes clear that tuning the many involved control parameters of this complex model is a tedious task, which requires prior optimizations with computationally high efforts. It still remains unclear how each parameter or even the CPG and reflex component in general contribute to stable and adaptable limit cycle motions. In which scenario is each of the two control approaches more beneficial? And what parameters must be adapted in different situations, e.g., frequency or wave form of the control signal?

To better understand the contribution of reflexes and CPGs during highly-dynamic movements, the present paper quantitatively compares these two control mechanisms from a functional point of view. As simple example, we regard a single compliant leg hopping forward as a basic form of a highly-dynamic limit cycle motion. We further simplify the complex interplay of all sensory input and motor output for individual joints by harvesting from the recent findings of Santello et al. (2016) how the CNS reduces the dimensionality of motor control signals by synergies. As mentioned by Del Vecchio et al. (2019), such dimensionality reduction simplifies the size of the search space that must be covered. Research by Lakatos et al. (2013, 2014) has shown how this concept can be put into an algorithm to control compliant limit cycle motions in robots in an energy-optimal way (Stratmann et al., 2016b). Stratmann et al. (2016a, 2018) also showed that the same principle is likely used by the CNS. For the comparison of the bioinspired control strategies, we are specifically interested to see how increasing levels of sensory information affect the performance of biomimetic systems. Thus, we apply a pure CPG, an adaptive CPG shaped by sensory feedback, and a pure reflex controller in the one-dimensional control space to the biomimetic robotic leg. We evaluate the performance of the biomimetic leg under each control approach in varying environment conditions regarding performance measures that are essential for the evolution of mammalian motion control, namely, stability and energy efficiency. To better understand the causes of the observed effects under the different control strategies, we study the system with increasing nuisance factors, i.e., stronger disturbances and higher loads. In the first step, we analyze the system by means of a multi-body simulation, in which the control signals are applied in an idealized manner. Following, the controllers are applied to a corresponding hardware setup including effects of actuator dynamics, realistic noise, and further constraints of real mechanical systems.

The contribution of the research presented in this paper is manyfold: First, we identify how reflexes and CPGs contribute to control highly-dynamic movements under different environmental conditions. This offers a guideline for future work to develop a strategy for bioinspired robots that unites the advantages of reflexes and CPGs, which can possibly improve current approaches for locomotion control. Second, the proposed method of comparison provides a benchmark case to which newly developed bioinspired control strategies can be compared to. Third, our results imply that adaptive CPGs must carefully adapt their oscillation shape to create meaningful body motion, which suggests a reevaluation of current mathematical CPG models that are usually designed to adapt only the control signal frequency. Fourth, due to the functional similarity of the tested controllers and biomimetic system to the biological counterparts, the findings might be able to trigger new bioplausible hypotheses about when reflexes and CPGs are active in mammals.

## 2. Method

To quantify the benefits that different biological control concepts provide during highly-dynamic compliant movements in changing environmental conditions, bioinspired controllers with increasing levels of sensory feedback were implemented to command a biomimetic robot leg. The system was analyzed in idealized simulation conditions as well as in experiments with corresponding robotic hardware to better understand the influence of each controller. In both cases, two changing environmental influences were applied to quantify the controllers' performance: First, we studied a fall from varying heights to analyze the controller's ability to recover to a limit cycle. Second, additional loads were applied to judge how efficiently each controller could insert energy into a system with changing dynamics. In the following, the model of the robot and the controllers are explained and the simulation and hardware setup are introduced. Additionally, the applied conditions and the metrics used for comparison are defined.

### 2.1. Biomimetic Robot Leg

**Figure 1:**
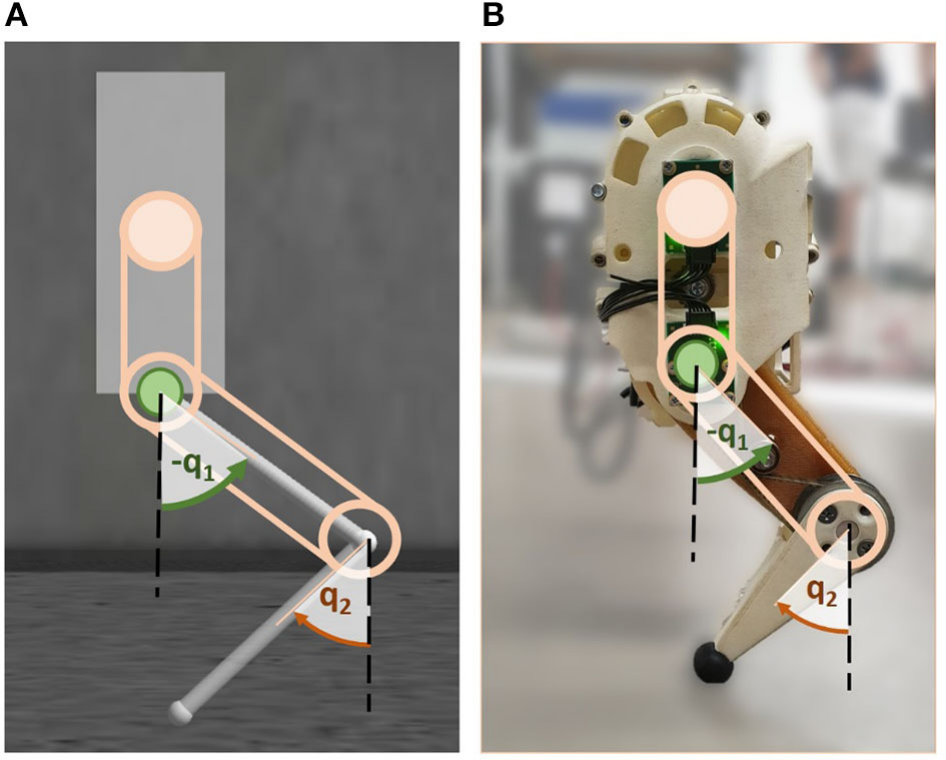
Stability and energy efficiency are analyzed by means of a biomimetic robotic hopper in simulation **(A)** and hardware **(B)**. The hopper consists of a floating base with two links connected by compliant joints. The hip (*q*_1_) and knee joint (*q*_2_) are independently driven by series elastic actuators, implying that the rotation of either joint does not affect the other joint. The beige circle indicates the knee motor location, the green one the hip motor. Angles are defined clockwise relative to vertically extended links.

Especially during highly-dynamic movements, the CNS of mammals must efficiently orchestrate the coordination of multiple joints of one limb and be able to quickly react to external changes. To understand the underlying control principles that allow this joint coordination, this work regards a simplified scenario of one single biomimetic robotic leg during hopping. The leg chosen as test platform is part of the compliant quadruped robot Bert (Lakatos et al., 2018) developed at the German Aerospace Center (DLR). It had already served as test bed for investigations on compliant mechanisms and bioinspired control previously (Lakatos et al., 2015; Stratmann et al., 2016b) and will allow for easy extension of the investigated concepts on locomotion in the future. The investigated control principles were applied to the leg in an idealized multi-body simulation implemented in Gazebo (cf. Figure 1A, section 2.1.1) as well as in a corresponding hardware setup to take into account realistic nuisance (cf. Figure 1B, section 2.1.2). In both cases, the upper body of the individual leg was considered to be a floating base that can freely translate, but was fixed in all rotational axes. This assumption was made because, as part of a complete (biological) quadruped, a leg is typically likewise constrained through the attachment to the trunk.

The floating base position was described by $x_{b}={\left(x_{b1},x_{b2}\right)}^{T}\in {\mathbb{R}}^{2}$. The leg attached to the base was composed of two serial links both with a length of 0.08 m connected by rotational compliant joints. The revolute joint between the base and the upper link was denoted as *hip*, while the connecting joint between the upper and lower link was defined to be the *knee*. The joint angles are denoted *q*_1_ and *q*_2_ for the hip and knee, respectively, and directions were defined as shown in Figure 1. The initial position of the system was defined to be at $q_{0}={\left(-0.7,0.7\right)}^{T}$ rad. The system coordinates are summarized by $x={\left({x_{b}}^{T},q^{T}\right)}^{T}\in {\mathbb{R}}^{4}$.

The two joints can be moved independently, each individually driven by a serial elastic actuator (SEA) with a stiffness *k*. The SEA of the hip was directly connected to the upper link, while the SEA for the knee was fixed in the upper part of the base and linked to the lower link via belt drives (cf. Figure 1). Deviating the SEAs in each joint by an angle θ_1, 2_ generated a torque in the corresponding joint. The algorithms that derive the control signal ***θ*** will be explained in detail in section 2.2. The complete dynamics of the system can be summarized by


(1) $$\begin{array}{l} M(x)\ddot{x}+C(x,\dot{x})\dot{x}+g(x)=\left(\begin{array}{c} 0\\ k(\theta -q)-c\dot{q} \end{array}\right)+\tau_{contact} \end{array}$$

where ***M***(***x***) denotes the symmetric positive inertia matrix, ***C***(***x***, ***ẋ***) the generalized Coriolis and centrifugal matrix, and ***g***(***x***) the gravitational forces. The generalized external forces are summarized by ***τ***_*contact*_. For more details refer to Lakatos et al. (2018).

#### 2.1.1. Simulation

As a platform for the idealized multi-body simulation of the system, the Neurorobotics Platform (NRP) developed within the Human Brain Project (Albanese et al., 2017) was chosen. Using the NRP allows the implementation and comparison of the technical bioinspired controllers with more sophisticated biological counterparts in future research. The underlying simulation framework used by the NRP was Gazebo 9.8.0 with ROS melodic. The NRP integrates a Python-based closed-loop engine that synchronizes the physics simulation with the high-level control implemented in Python after each global NRP timestep (Hinkel et al., 2017). As simulation parameters, the NRP default settings were used with a time step of 0.001 s for the Gazebo physics engine and 0.02 s as global NRP step to update the controller models.

The robot was implemented as a multi-body system according to the above explained definitions. The floating base is constrained accordingly in all rotation axes and only able to translate vertically and horizontally. In line with previous simulations of the biomimetic leg carried out by Stratmann et al. (2016b), the mass of the floating base was set to 0.49 kg. The masses of the upper and lower leg link were set to 0.059 and 0.038 kg, respectively. The stiffness *k* for the SEA was set to be 1.46 N m rad-1. Additionally, a damping factor of *c* = 0.0219 N m s rad-1 was added in the two joints to account for friction occurring in the real robot. The friction between the robot foot and the ground was defined as μ = 1.0. Researchers who want to replicate the simulation can find the model as template experiment on the openly accessible NRP^1^. For the idealized simulation scenario, control signals of the different controllers were applied instantaneously as joint torques without taking into account motor dynamics or other delay.

In each simulation, the robotic leg was initialized at a base height of 20 cm above the ground or obstacle. At simulation start, the hopper dropped and started jumping forward driven by one of the investigated controllers. All simulations were repeated 10 times to average out non-deterministic effects due to synchronization of the different sampling times in the NRP.

#### 2.1.2. Hardware

**Figure 2:**
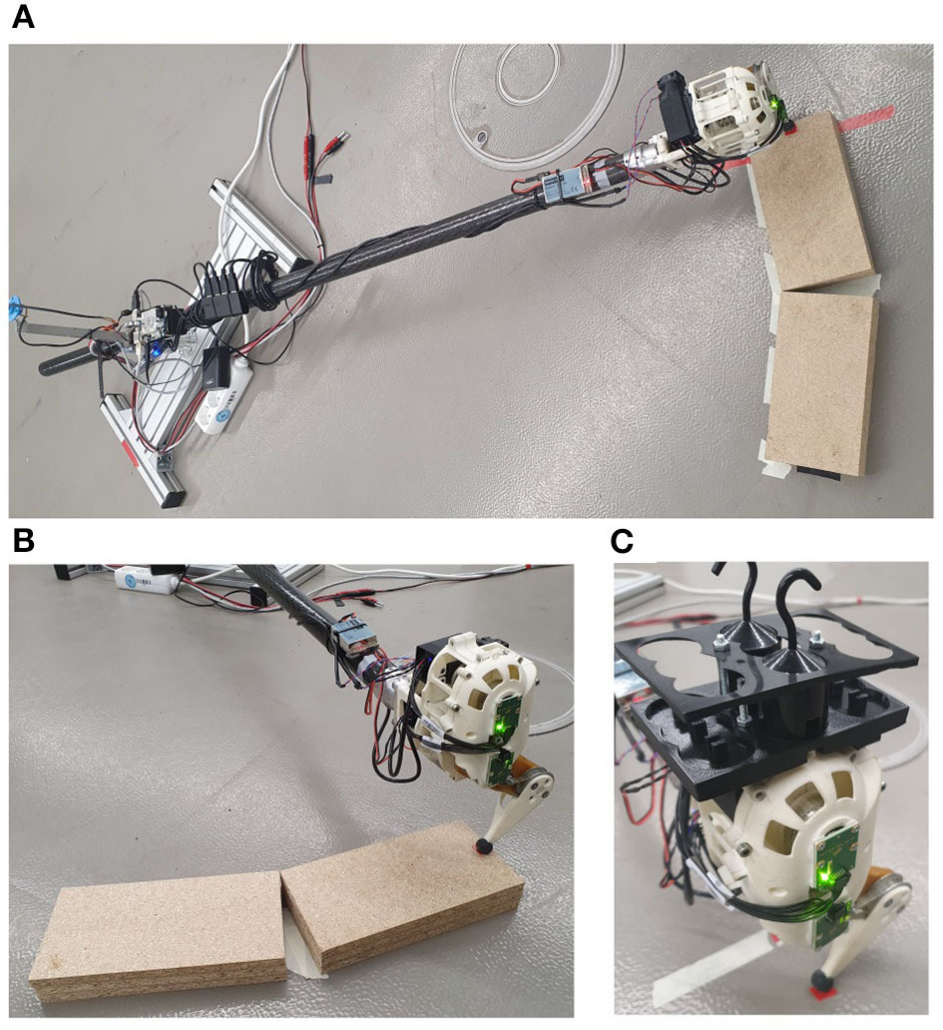
**(A)** In the experimental hardware setup, the robotic hopper is attached to a boom to realize the floating base. **(B)** In the fall scenario the hopper is initialized on a plateau. The gap between the plateau pieces did not cause any disturbance. **(C)** For the analysis of energy efficiency, an increasing load in form of weights is attached to the system.

For the hardware experiments, the biomimetic robotic hopper described by Lakatos et al. (2018) was used. It had a total mass of 0.562 kg and the SEA stiffness was measured to be *k*≈2 N m rad-1. To realize the mentioned rotational constraints, the base was attached to a boom so that it could freely jump vertically and move horizontally in a circle around a fixed center (cf. Figure 2A). Due to a limited range of motion in the physical system, the defined initial position of the joints was adjusted to $q_{0}={\left(-0.61,0.61\right)}^{T}$ rad. Sensors in each joint measured the link positions *q* and motor positions ***θ***. A sensor in the boom joint measured jump height and horizontal position.

For the control and communication infrastructure it was chosen to use proprietary DLR software, despite the fact that the NRP had been designed to easily replace a robot simulation with corresponding hardware. This feature makes the NRP a sophisticated tool to investigate bioinspired control strategies, especially when considering bioplausibe neuron models, but in the present line of research we were not able to take advantage of this characteristic. Since the proprietary communication infrastructure had already been implemented with the robot and was tested to be robust, it was preferred over a new integration with the ROS interface that is used by the NRP. While this entailed that the investigated controllers had to be coded twice, the implementations were mathematically equivalent for the simulation and the hardware experiments and the results are thus comparable. For the experiments, the bioinspired controllers were implemented in a Matlab Simulink model, which communicated with the hardware in a 1 kHz control cycle. The commanded and measured link and motor positions, as well as the boom sensor measurements, were also recorded at 1 kHz. The low-level control of each individual SEA motor was realized through PD-control, where the proportional term had a gain of *P* = 55 and the derivative term was set to zero in the experiments.

At the beginning of the experiment, the joint sensors were calibrated once, while the boom sensor was calibrated before each new test condition. For both calibrations, the robot legs were extended straight down, such that ***q*** = (0, 0)^*T*^. At the beginning of each trial, the system was placed at the same position indicated by a marker on the floor (cf. Figure 2C). The experiment was started from a resting equilibrium position. To ensure repeatability, each trial was repeated three times.

### 2.2. Controller Design

For high-level control, different bioinspired controllers with a varying balance between intrinsic CPG-based oscillation and reflexive sensory feedback were implemented. Established control models were used that can be regarded as simplified implementations of the biological principles reflecting the main characteristics of reflex control and CPGs as well as a combination of both.

#### 2.2.1. Control Space

As previously mentioned, this work leverages the findings by Santello et al. (2016) that the mammalian CNS reduces the dimensionality of motor control signals. Recent insights gained through simulations (Stratmann et al., 2016a) support this hypothesis, possibly pointing to a pathway in the CNS that realizes such a dimensionality reduction. Such a reduction of sensory input of multiple joints to a lower-dimensional control space simplifies the control problem and reduces the parameter space to be considered. Simultaneously, robotic experiments with a functionally analog dimensionality reduction have shown that essential motion properties such as movement stability (Lakatos et al., 2013) and energy optimality (Stratmann et al., 2016b) are maintained with this approach.

In this work, the transformation from the multi-DOF joint space to the 1D control manifold and back to the joints was carried out by means of an adaptable weight vector *w* in IR^n^, where *n* denotes the number of actuated joints. This allows in the first step the reduction of the sensory input from all joints, i.e., the joint torques ***τ***, to a combined scalar feedback signal,


(2) $$\begin{array}{l} \tau_{z}=\frac{w^{T}}{\left||w|\right|}\tau \;. \end{array}$$

This feedback signal τ_*z*_ combines information about all joints and can be used to derive the control signal θ_*z*_ according to the different bioinspired control strategies (reflex or CPG) as described in the following subsections. The one-dimensional control signal is then mapped back to the joint space using the same transformation weights according to


(3) $$\begin{array}{r} \theta =\frac{w}{\left||w|\right|}\theta_{z}\;. \end{array}$$

The signal θ_*i*_ is then commanded to the corresponding *ith* joint to drive the joint motion. Thus, the implementation of the different bioinspired control mechanisms in the one-dimensional control space allows the quantifiable comparison of the investigated strategies. Here, the weights were set constant to focus on the effect of the different control strategies. Previous work (Lakatos et al., 2013; Stratmann et al., 2016b) described how to choose and adapt the modal weights ***w*** to changing environments.

#### 2.2.2. Reflex Control

A pure reflex forwards sensory information directly toward motor output. In mammals, this reflex is usually triggered by cutaneous or load receptors (Bastiaanse et al., 2000; Nielsen and Sinkjaer, 2002) and has also been implemented in this way in different robots using contact sensors to trigger the control signal (Manoonpong et al., 2007; Zhao et al., 2020). Nevertheless, this implementation approach was not used in this work, mainly due to hardware limitations at the point of experimentation, where a contact sensor was not available. Instead, we used the approach previously presented by Lakatos et al. (2013), in which a bang-bang controller was triggered when crossing a certain torque threshold in the joints. However, the concept of using either a contact sensor or the joint torques to trigger the reflex response are functionally equivalent, because the joint torques proportionally depend on the contact forces during stance phase. For our implemented reflex controller, the joint torque is a function of the motor coordinate ***θ*** and the joint state *q* and depends on the stiffness ***k*** of the SEA spring, which was defined in section 2.1:


(4) $$\begin{array}{r} \tau =k\left(\theta -q\right)\;. \end{array}$$

The torque is transformed from the higher dimensional joint space, onto the 1D control signal according to Equation (2). The combined feedback signal τ_*z*_ is then compared to a threshold value ϵ_τ_ to trigger the control signal θ_*z*_ of constant amplitude:


(5) $$\begin{array}{r} \theta_{z}=\{\begin{array}{ll} +\;\hat{\theta_{z}} & \;if\;\tau_{z}>\epsilon_{\tau }\\ \;0 & \;if\;-\epsilon_{\tau }\leq \tau_{z}\leq \epsilon_{\tau }\\ -\;\hat{\theta_{z}} & \;if\;\tau_{z}<-\;\epsilon_{\tau } \end{array}. \end{array}$$

The control signal θ_*z*_ is then mapped back into joint space as described in Equation (3) and applied to drive the position of the SEA in each joint. It is important to note that in contrast to the work of Lakatos et al. (2013) the torque threshold was tuned in such a way that it was not crossed during the flight phase, thus only triggering the reflex during the stance phase.

In order to prove that the chosen approach to implement a reflex is indeed functionally equivalent to the control method based on contact sensing, for the simulations we additionally implemented the reflex as proposed by Zhao et al. (2020). As described in Equation 9 of their paper, we triggered the control signal θ_*z*_ based on perceived ground contact plus a given time delay. We exemplary carried out the simulations for the fall condition with this reflex trigger to show that both approaches lead to the same fundamental behavior. All other simulations and the experiments were carried out using the method based on Lakatos et al. (2013) described by Equations (4) and (5).

#### 2.2.3. CPG Control

To replicate the working principle of a CPG, we implemented the control model proposed by Matsuoka (1985) and Matsuoka (1987), which is one of the most widely used methods in robotics and computational neuroscience (Yu et al., 2013). It implements two reciprocal inhibiting oscillators, of which one is usually controlling an extensor unit, the other one a flexor unit. While the investigated leg model is not actuated by antagonistic actuators, the flexor unit is active when the leg lowers down to the ground and the extensor unit drives the upwards motion to jump. Each oscillator is governed by the following set of equations:


(6) $$\begin{array}{l} \begin{array}{r} \begin{array}{l} \tau_{u}{\dot{u}}^{\left\{e,f\right\}}=-u^{\left\{e,f\right\}}+w_{fe}y^{\left\{f,e\right\}}-\beta v^{\left\{e,f\right\}}+u_{0}+{\text{Feed}}^{\left\{e,f\right\}}\\ \tau_{v}{\dot{v}}^{\left\{e,f\right\}}=-v^{\left\{e,f\right\}}+y^{\left\{e,f\right\}}\\ \;y^{\left\{e,f\right\}}\;=\text{max}(u^{\left\{e,f\right\}},0)\\ \;\theta_{z}\;=-y^{\left\{e\right\}}+y^{\left\{f\right\}}\;. \end{array} \end{array} \end{array}$$

The superscripts *e* and *f* correspond to the extensor and flexor unit, respectively. The outputs *y*^{*e,f*}^ of the two units depend on the inner state *u*^{*e,f*}^ and the degree of self-inhibition *v*^{*e,f*}^ in each unit scaled by β. The connection strength of the two oscillating units is defined by *w*_*fe*_. Through the tonic input *u*_0_ a constant drive is induced to the CPG. For a pure CPG control, where the signal is not shaped by feedback of the system, the variable Feed was set to zero. To take into consideration the control possibility in which the CPG is adapted by means of sensory feedback, the Feed variable can be added. This combines the influence of the feedforward CPG approach with the state-based reflex. To allow a valid comparison with the reflex, the previously mentioned scalar feedback variable τ_*z*_ was used here as state variable of the mechanical system. The degree of entrainment with the sensory feedback could be adjusted by the gain *h*.


(7) $$\begin{array}{l} {\text{Feed}}^{\left\{e\right\}}=-{\text{Feed}}^{\left\{f\right\}}=\{\begin{array}{l} h\;\tau_{z}\;if\text{ ‘CPG+sensory input'}\\ \;0\;if\text{ ‘pure CPG' } \end{array}. \end{array}$$

Due to the reciprocal inhibition, the feedback of the two oscillation units needed to be opposite in sign. Identical to the reflex controller, the 1D control signal θ_*z*_ that is commanded in the latent space was transformed according to Equation (3) to drive the SEA in the knee and hip joint.

### 2.3. Controller Tuning

With the above mentioned methods, three bioinspired control algorithms were implemented on the biomimetic leg in this work: (1) the reflex representing control solely dependent on sensory information, (2) a CPG including feedback to shape the control signal, thus functioning as adaptive oscillator, and (3) a pure CPG without sensory feedback to completely exclude sensory modulation of the intrinsic CPG oscillation (nF-CPG).

The inclusion of feedback means that the reflex and adaptive CPG will automatically adjust to match the inherent frequency of the mechanical system. However, the nF-CPG needs to be tuned manually to match the intrinsic dynamics of the mechanics. Only then can a fair comparison between the different control strategies be drawn. To achieve this, all controllers were empirically tuned to obtain a similar hopping trajectory for the default system without applied environment changes. The corresponding parameter values for all controllers are shown in Table 1. With these parameters, a visually similar hopping motion of the biomimetic leg could be generated with all implemented bioinspired control methods in both the simulation and the hardware setup. In simulation, the biomimetic leg was hopping with a similar frequency with all applied bioinspired control strategies. The pure reflex controller and the nF-CPG led to a hopping frequency of 3.12 and 3.13 Hz, respectively, while the frequency with the modulated CPG was 3.2 Hz. As previously mentioned, to prove functional equivalence of our reflex implementation with the more bioplausible method that triggers the control signal based on contact sensing, we additionally implemented and tested the method of Zhao et al. (2020) for the fall condition in simulation. The time delay for the trigger after ground contact detection was set to the minimum possible value, i.e., 0.02 s, which is the used default time step of the NRP. The control signal θ_*z*_ as well as the weighting parameters remained unchanged. With this reflex controller the identical hopping frequency of 3.12 Hz as for the torque triggered reflex was achieved.

**Table 1:** Parameter values used for the tested controllers.

**Reflex**		**CPG**		**nF-CPG**	
**Param**	**Value**	**Param**	**Value**	**Param**	**Value**
$\hat{\theta_{z}}$	±0.45 (0.23)	τ_*u*_	0.04	τ_*u*_	0.045
ϵ_τ_	0.3	τ_*v*_	0.08	τ_*v*_	0.09 (0.08)
		β	2.5	β	2.5
		*w*_*fe*_	-2	*w*_*fe*_	-2
*w*_1_	-0.5	*u*_0_	0.4 (0.2)	*u*_0_	0.6
*w*_2_	1.0	*h*	0.3	*h*	0

*The values in brackets differed in the hardware setup from the simulations. Weighting w_1, 2_ is applied to all controllers*.

In hardware, the hopping frequency with the unmodulated nF-CPG resulted in 3.05 Hz, while the hopping with the feedback adapted controllers both resulted in a frequency of 2.90 Hz.

### 2.4. Environmental Conditions

To investigate the influence of the different bioinspired control strategies with regard to stability and energy efficiency, two environmental conditions were considered. In each experiment trial, a total of 20 s was recorded and analyzed.

The first condition aims to investigate the ability of the controlled system to return to a limit cycle after a disturbance. For this purpose, the hopper robot was dropped from a plateau of varying heights. The hopper was initialized on top of the plateau at the beginning of the experiment, hopping forward on the plateau for approximately 10 s to achieve a limit cycle motion. Subsequently, the robot reached the end of the plateau and dropped forward to the ground, causing a horizontal and vertical disturbance at the same time. The experiment was recorded for another 10 s to give the hopper a sufficient amount of time to recover from the fall and return to a stable limit cycle. In the simulation setup, the experiment was carried out for ten different plateau heights starting from 2 cm and increasing to 20 cm in steps of 2 cm. For the hardware setup, the experiment was carried out solely for plateau heights between 1 and 5 cm to protect the hardware from mechanical damage. In each trial, the height was increased by 1 cm (cf. Figure 2B).

In the second environmental condition, the ability of each bioinspired controller to react to increasing load and efficiently apply energy to the robotic system during highly-dynamic motions is explored. For the simulation, a sudden onset of load was applied after 10 s of undisturbed jumping. Eleven different cases were considered, applying additional load forces from 0 to 5.5 N in increments of 0.5 N. For hardware validation, 54 g were added to the system initially. In each of the following five trials, the load was repeatedly increased by 100 g (cf. Figure 2C). Thus, up to 200 % of the hopper's initial gravitational force were applied as additional load in the simulations and the hardware setup.

### 2.5. Performance Metrics

For the quantified comparison of the tested bioinspired controllers, the stability and energy efficiency of each controller was analyzed. For this purpose, two performance metrics were defined.

**Figure 3:**
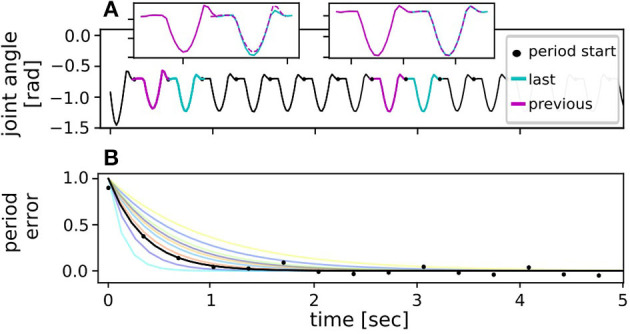
Data analysis to derive stability metric. **(A)** To find the convergence errors for the stability metric, the joint trajectory in each (last) period was compared to the previous one by autocorrelation, where a period was defined to start at the maximum body height in flight phase. The overlayed trajectories of the two periods were subtracted resulting in a cumulative error value per period. **(B)** The cumulative error value determined for every period was expected to decrease with time after a disturbance and reach zero once the hopping leg returned to a limit cycle motion. To better compare the convergence properties of the different fall heights, the error curves were normalized for the different test conditions and then fitted with an exponential curve over all repetitions. Exemplary data (black) are shown for a simulated fall from 12 cm while being controlled by the reflex. Curve fits for all other fall heights are shown in other colors.

The first metric was dedicated to judge the ability of each controller to recover from the fall disturbance and return to a stable motion. Common limit cycle metrics such as a Poincaré map were not used, as they consider only individual points across the movement cycle. To better judge the overall trajectory progression, instead a metric based on autocorrelation of the state variables was chosen. For this, the joint trajectory in each period was compared to the trajectory in the previous period (cf. Figure 3A). The curves of the two periods were overlayed with maximum correlation and then subtracted from each other. The mean difference of the overlayed trajectories within two subsequent periods was defined to be the convergence error in every period. This measure quantifies the deviation from the limit cycle. Right after a disturbance, the convergence error should thus be large as the joint trajectories of two following periods do not match. Once stable periodic motion is reached again, the error should converge to a constant value, ideally zero. The overall error curve was hypothesized to decay exponentially after the initialized disturbance, which indeed fitted the data very well (cf. Figure 3B). Thus, by using the least square method an exponential curve was fitted through the error values for every tested disturbance condition:


(8) $$\begin{array}{l} e_{\text{conv}}\left(t\right)=E_{0}\;e^{-\lambda t}+e_{\infty }\;, \end{array}$$

where the exponent λ determines how fast the error signal decays, i.e., how fast the applied controller can return the system to stable limit cycle motion. The constant *e*_∞_ corresponds to the final converged error value. In theory this value should be zero during limit cycle motion with the joint trajectories of every period being identical. However, slight offsets were possible in the considered test case as e.g., the hardware environment poses non-deterministic effects. The fitting parameter *E*_0_ describes the initial error magnitude at the moment of disturbance. However, since the biomimetic leg did not only fall down, but simultaneously fell forward during the stability testing, the initial error magnitude did not scale proportionally with the tested fall heights. Instead, error decay λ characterized each controller's ability to return the system to a limit cycle motion. Consequently, for better comparison of the bioinspired controllers in the different tested conditions, i.e., fall heights, the error curves were normalized by subtracting the converged end value and dividing by the peak value such that *E*_0_ = 1 and *e*_∞_ = 0. For a more intuitive expression, the error decay in each condition could be expressed in terms of half-life *T*_1/2_ time by


(9) $$\begin{array}{r} \lambda =\frac{ln\left(2\right)}{T_{1/2}}\;. \end{array}$$

The half-life times in all conditions were statistically compared between the different controllers with a paired *t*-test, by accessing the simulation and hardware setup separately.

As metric for the energy efficiency, the amount of energy the motors inserted per limit cycle was compared to the amount of internal energy in the system during each period. This measure was chosen instead of conventional measures such as Cost of Transport (COT), which relates energy to traveled distance. However, for this work the COT was regarded as insufficient metric, since the goalwas not to judge the efficiency in terms of locomotion capabilities, but focused on the controllers' ability to efficiently add energy during highly-dynamic motions. The energies were calculated for each period individually over 10 s. A period was defined to lie between two peak points of the body height when jumping in a stable limit cycle. For each period the motor energy was calculated by


(10) $$\begin{array}{l} E_{\theta }=\int_{\theta }k\left(\theta -q\right)\;d\theta \;. \end{array}$$

The positive energy input $\left\lceil E_{\theta }\right\rceil =E_{\theta }^{+}$ in the two joints of the system corresponds to the total energy added by the controller. Negative energy values $\left\lfloor E_{\theta }\right\rfloor =E_{\theta }^{-}$ indicate that the motor torque was commanded with the same direction as the joint velocity, which pulled the motor to essentially act as a generator. This energy was considered to be “lost,” neither animals nor most robots can recuperate energy. The internal energy of the mechanical system is estimated at the peak point of the body in each period. The jumping height *h* is set relative to the equilibrium position of the resting body with the respective load applied for the tested condition. The total energy of the system *E*_*sys*_ at that point is then calculated by


(11) $$\begin{array}{r} E_{sys}=m_{tot}gh+\frac{1}{2}kq^{2}+\frac{1}{2}m_{tot}v^{2}. \end{array}$$

Where *m*_*tot*_ is the total system mass including the applied load, and *v* denotes the system's full velocity at that point. At the peak point of the flight phase, the rotational kinetic energy is expected to be negligibly small, while the majority of the system's energy comes from the potential height energy. Unlike mechanical efficiency, the resulting energy ratio κ between internal system energy and motor energy can be >1 as energy in the system can accumulate over multiple cycles. The energy metric κ for each controller is defined by


(12) $$\begin{array}{r} \kappa =\frac{E_{sys}}{E_{\theta }}. \end{array}$$

## 3. Results

In simulations and hardware experiments, different bioinspired controllers were implemented to drive a biomimetic compliant robot leg hopping forward as example of a highly-dynamic movement. The controllers were tested in two changing environmental conditions to compare their stability against disturbances as well as the energy efficiency of the excited movements. In the following, first the controllers' ability to recover a stable motion after a fall is described. Subsequently, the efficiency of each controller to insert energy into the system under different loads is presented.

### 3.1. Stability

**Figure 4:**
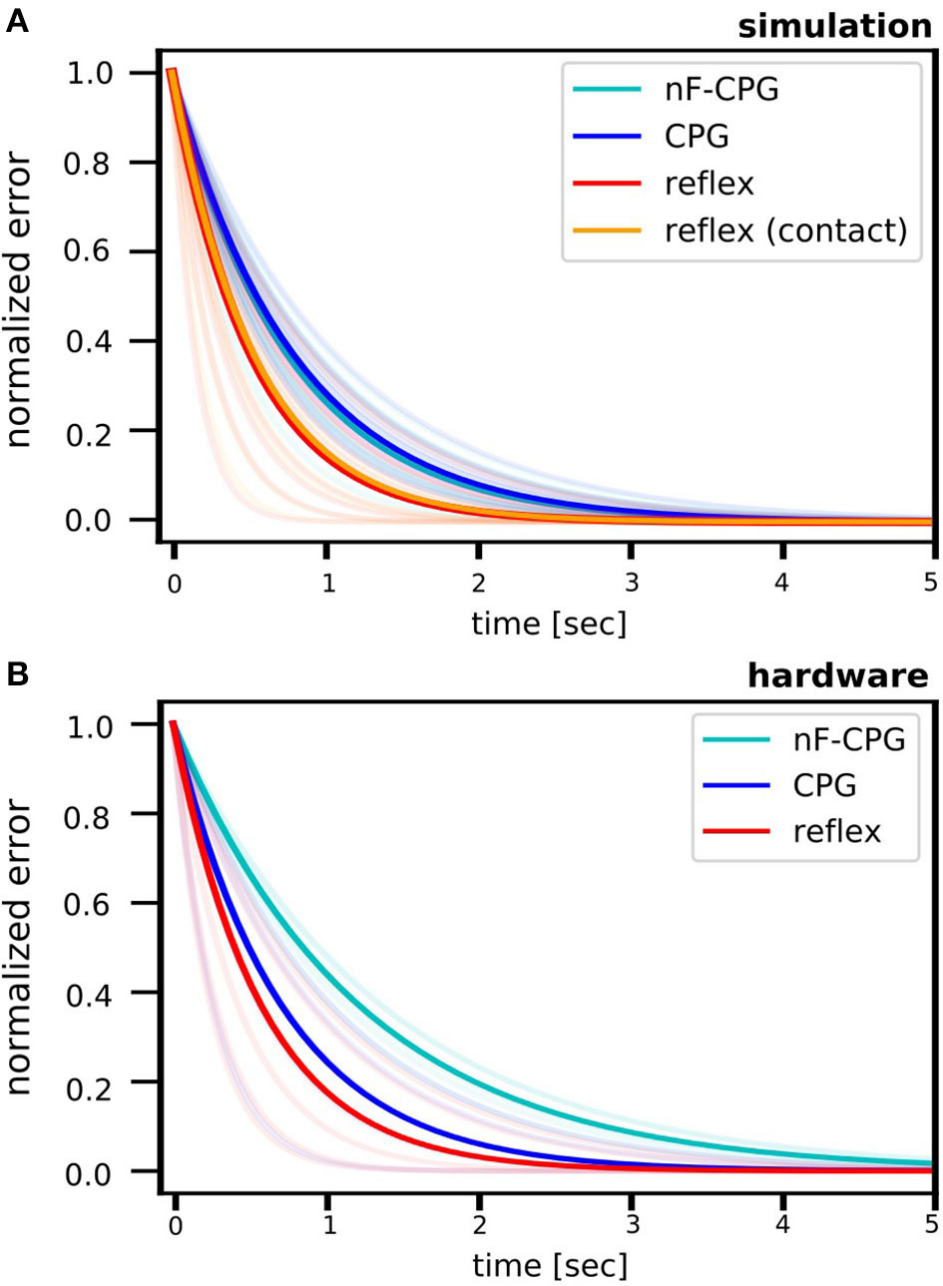
Fitted curves for the cumulative errors determined per period in the fall scenario for the biomimetic leg. Results are shown for the simulation **(A)** and the hardware setup **(B)**. For better comparison, all error curves were scaled by the initial disturbance value at the fall (*t* = 0), thus all starting at 1. The light-colored curves show the individual fits for each fall height. Overall, the reflex was fastest to recover a stable motion. In the simulation, the reflex was tested with two different methods, either triggered through (1) contact sensing (orange) or (2) a torque threshold (red). As expected by theory, no difference between the two trigger methods could be found, thus, validating functional equivalence. While triggering based on ground contact is the bioplausible strategy, we chose the latter to implement the reflex on the robotic leg in hardware without the need of additional sensors.

Analyzing stability showed that all tested controllers were able to recover the biomimetic leg to the hopping limit cycle motion after the disturbance through a fall (cf. Figure 4). In the simulations and hardware experiments, the reflex controller was overall fastest to converge back to a stable motion.

Over all conditions, in the simulation the reflex converged significantly faster back to a stable limit cycle than the adaptive CPG [0.56 ± 0.05 s (ste); *p* = 0.008] and the CPG without feedback modulation [nF-CPG, 0.53 ± 0.04 s (ste); *p* = 0.021]. As expected by theory, it showed to be irrelevant if the reflex was triggered through contact sensing [0.38 ± 0.05 s (ste)] or the torque threshold [0.37 ± 0.03 s (ste)]. Between both methods, no significant difference was found and an overlaying curve progression of the stability measure was obtained (cf. Figure 4A). This proves the functional equivalence of the two reflex implementations. Thus, in the following, we will solely compare and refer to the torque-triggered reflex controller.

**Figure 5:**
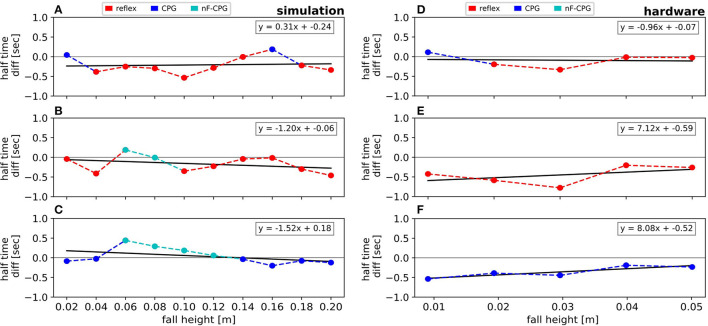
Visualization of the half-life time differences between the reflex, the adaptive CPG and the pure (no feedback) CPG as stated in Table 2. Results are shown for the simulation **(A–C)** and the hardware experiments **(D–F)**. The half-life times are individually compared for the reflex vs. the adaptive CPG **(A,D)**, the reflex vs. the pure CPG **(B,E)** and the adaptive CPG vs. the pure CPG **(C,F)**. The colors are chosen according to which controller was faster in each condition. For every comparison a curve is fitted to investigate the trend.

Figures 5A,B emphasizes the faster recovery of the reflex in comparison the two CPGs for each fall height individually. Averaged over all fall heights, the two CPGs showed a similar performance (cf. Table 2). However, a closer look at the individual half-life times shows a notable trend (cf. Figure 5C): for falling heights below 14 cm, the nF-CPG had a similar if not better ability to converge back to a stable motion in comparison to the adaptive CPG. But above 14 cm, this trend seemed to flip to the opposite and the adaptive CPG started to recover faster than the unmodulated CPG.

**Table 2:** Error half-life times (in seconds) for the different fall disturbances in simulation and hardware; last line corresponds to the fit through all condition errors.

	**Simulation**			**Hardware**		
**Height**	**Reflex**	**CPG**	**nF-**	**Reflex**	**CPG**	**nF-**
****[**cm**]****			**CPG**			**CPG**
1				0.3	> 0.18	<0.72
2	0.4	> 0.36	<0.44	0.2	<0.39	<0.78
3				0.17	<0.5	<0.95
4	0.18	<0.57	<0.59	0.62	<0.64	<0.83
5				0.69	<0.72	<0.95
6	0.5	<0.76	> 0.31			
8	0.37	<0.66	> 0.37			
10	0.11	<0.64	> 0.46			
12	0.24	<0.53	> 0.47			
14	0.44	= 0.44	<0.48			
16	0.66	> 0.47	<0.67			
18	0.37	<0.6	<0.67			
20	0.28	<0.62	<0.74			
Overall	0.37	<0.56	> 0.53	0.4	<0.5	<0.85

The averaged half-life times in the hardware closely matched the overall results of the simulations for the reflex with 0.4 ± 0.06 s (ste) and the adaptive CPG with 0.5 ± 0.07 s (ste). Although the reduced number of trials did not allow statistically significant conclusions, the individual conditions (cf. Figures 5D–F and Table 2) showed a similar trend with the reflex usually being fastest to recover the limit cycle motion. The nF-CPG recovered the limit cycle with 0.85 ± 0.15 s (ste) overall much slower than in the simulation and significantly slower (*p* = 0.003) than both feedback modulated controllers in the hardware (cf. Figures 5E,F).

### 3.2. Energy Efficiency

**Figure 6:**
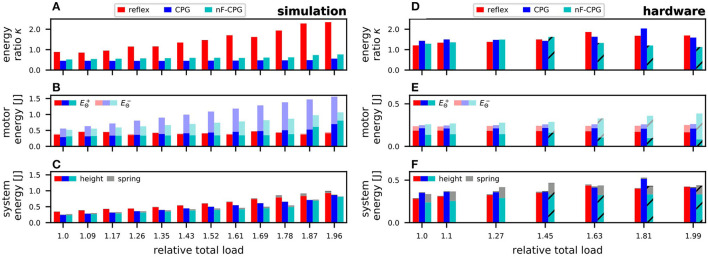
Comparison of the energies and their ratios to judge the energy efficiency of the different controllers. Results are shown for the tested load conditions in simulations **(A–C)** and corresponding hardware setup **(D–F)**. **(A,D)** The energy ratio κ quantifies the controllers' ability to efficiently add motor energy to the mechanical system. In the simulations, the reflex outperformed both CPGs, while in the hardware similar results were obtained. **(B,E)** The effective energy generated by the motor $E_{\theta }^{+}$ was mainly constant while the different controllers appeared to “lose” a varying proportion of motor energy ($E_{\theta }^{-}$), especially in the simulations. **(C,F)** The internal system energy was mainly dependent on the relative jump height. Only for the nF-CPG in the experiment did a higher proportion relate to spring deflection (gray). The x-ticks indicate the total system mass relative to the initial mass without load, with the first tick having no load attached. The striped bars indicate trials in which the mechanical system could not hop anymore.

To assess how well each bioinspired controller could insert energy in the biomimetic leg during hopping as an example of a highly-dynamic movement, the introduced energy metric was analyzed. For this purpose, the energy stored within the robotic system was calculated by Equation (11) and set in relation to the energy inserted by the controllers in each step (cf. Equation 10). The higher the resulting energy ratio κ was, the more efficiently a controller could inject energy into the system. In the following, the results of the energies are reported separately for the simulation (cf. Figures 6A–C) and the hardware experiments (cf. Figures 6D–F).

As illustrated by the higher energy ratio κ in Figure 6A, in simulation the energy efficiency for the reflex controller was highest over all load conditions. With more load, the reflex even seemed to become more efficient. In contrast, the energy ratio κ for both CPGs was relatively constant with no significant difference between them. To better understand these energy ratios, the two influencing factors, (1) energy inserted by the controller, and (2) internal system energy, are examined in the following.

**Figure 7:**
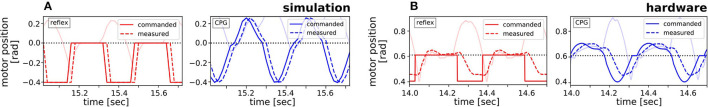
Comparison of the commanded (solid line) and measured (dotted line) motor signal for the reflex (red) and the adaptive CPG (blue), exemplarily shown for the knee without applied load. **(A)** In the simulation, the ideal curve could be followed. **(B)** In the hardware experiment the commanded signal was delayed through the motor dynamics leading to overall more similar control signals.

The inserted energy (cf. Figure 6B) showed a contrasting progression to the energy ratios: The inserted energy through the reflex stays constant over all loads, while the energy inserted by both CPGs increased seemingly linearly with higher loads. This related to the way the motor signal was commanded. While the reflex was applied instantaneously, the CPGs were commanded as a continuous signal (cf. Figure 7A). Thus, for both CPGs a notable amount of inserted energy was found to be “negative energy” ($E_{\theta }^{-}$), i.e., the motor essentially acting as a generator. This “lost” energy was particularly high for the adaptive CPG. However, the portion of the effectively usable motor energy ($E_{\theta }^{+}$) that was driving the hopping motion, did not show a significant difference between the reflex and any of the CPGs.

The corresponding internal energy, i.e., potential energy within the leg at the peak position of the flight phase (cf. Figure 6C) increased for all controllers over increasing loads. This was due to the fact that with increasing load the equilibrium position of the biomimetic leg likewise decreased. Relative to this new (lower) equilibrium position the jump height only decreased little. At the same time, the added weights caused the total mass *m* of the system to be substantially higher, which explains the overall increase in potential energy for all controllers.

In the hardware experiments, the energy ratios based on the inserted motor energy and internal system energy showed clear differences from the simulation results (cf. Figure 6D). The energy ratios of the reflex and the adaptive CPG were overall in a similar range independent of the applied load. The nF-CPG was unable to jump when more than 150 g were added, instead starting to teeter in place.

Individual examination of the inserted motor energies (cf. Figure 6E) showed that the reflex and the adaptive CPG were able to supply constant input for all loads with only a small portion being “lost” ($E_{\theta }^{-}$). Although the reflex was still commanded as an instantaneous signal, the dynamics of the motor caused the measured motor signal to be very similar for the reflex and the adaptive CPG (cf. Figure 7B). In contrast, the nF-CPG showed an increasing amount of “lost” energy, especially when it became unable to jump (above 150 g) and instead teetered in place. Thus, the effective motor energy $E_{\theta }^{+}$ inserted by the controllers with feedback was seen to be higher.

As for the system's total internal energy (cf. Figure 6F) it could be observed that it stayed relatively constant for all controllers over the increasingly higher applied loads. For the reflex and the adaptive CPG this is due to the fact that the increase in total system mass seemed to be proportional to the jump height relative to the decreasing equilibrium position. For the nF-CPG, however, it could be observed that the leg motion was generally not in sync with the control signal. Therefore, the joint springs did not return to their zero position at the body's peak point but were actively deflected. Thus, internal system energy was not solely due to the potential height energy, but a noticable amount was also due to the deflection of the springs at the peak point.

As expected, the controllers with feedback component could successfully adjust the signal frequency to the changing system behavior, while for obvious reasons the frequency of the nF-CPG remained unchanged. In favor of both CPGs, however, it must be mentioned that they were always able to initiate some sort of periodic motion in the biomimetic leg, even when it was only teetering in place. In contrast, the reflex controller fell silent when the load was further increased in the simulation as the needed threshold was not triggered.

## 4. Discussion

In order to better understand underlying control principles in the CNS for highly-dynamic motions, different bioinspired control strategies were investigated on a biomimetic robotic leg. We determined how increasing levels of sensory feedback influence two key motion performance measures under changing environmental conditions: stability and energy efficiency. As anticipated, the fall conditions showed that a pure reflex-based control most effectively supports inherent system dynamics, which leads to stable limit cycle motions and very fast recovery from disturbances. Without continuous feedback modulation, a quick recovery is only possible for small disturbances, even when a controller is well-entrained to the system. Applying different loads to the biomimetic system to study the energy efficiency also led to questioning the commonly held assumption that a controller needs only to adjust its frequency to match the inherent dynamics of a system (Buchli et al., 2006; Iwasaki and Zheng, 2006; Dzeladini et al., 2014; Khoramshahi et al., 2017; Santos et al., 2017). It seems equally important to adjust the temporal shape of the control signal in order to drive energy efficient motions.

With these findings, the presented work provides new insights with respect to existing literature. Previous research mainly focused only on analyzing benefits of individual biological control strategy, i.e., reflexes (Geyer and Herr, 2010) or CPGs (Dzeladini et al., 2018). Dzeladini et al. (2014) already presented early results showing how CPGs might extend reflexive pathways as feedback predictors. We here extend this work by a systematic comparison that quantifies the influence of bioinspired feedback and feedforward components on motion performance. Such a comprehensive overview has so far been missing, partly due to the fact that a meaningful comparison of different controllers is not trivial because of the large number of degrees of freedom of biological locomotor systems. In this paper, such a comparison was made possible by our recent work that showed how the brain can control complex compliant movements from a one-dimensional synergy manifold (Lakatos et al., 2013; Santello et al., 2016; Stratmann et al., 2016b; Del Vecchio et al., 2019). This work allowed the analysis of a large parameter space by implementing the different controllers in this low-dimensional control manifold.

Our comparative investigations led to three important contributions: First, we presented a setup with quantifiable metrics to identify the relevance of reflexes and CPGs in different environmental conditions. Second, we provide a useful benchmark to better compare the performance of different bioinspired controllers in the future. Third, our results point out possible ways to improve current control strategies for highly-dynamic motions in compliant robots, which could also benefit strategies for locomotion control. Forth, due to the biological plausibility of the control space concept (Stratmann et al., 2018) and applied control strategies, our findings can additionally help to generate testable hypotheses about control mechanisms in mammals.

### 4.1. Stability

The reflex controller is shown to be faster than both CPGs to converge the mechanical system back to a limit cycle motion after the fall disturbance. This is in line with previous findings on dynamic walking (Schwab and Wisse, 2001; Geyer and Herr, 2010; Maus et al., 2010) showing that pure feedback control can effectively support the stability of compliant systems and return them to a limit cycle motion after disturbance. The drawback of this control strategy, however, is that it is hard to modulate the gait pattern, i.e., tune the step length or speed (Dzeladini et al., 2014). Thus, it is commonly hypothesized that a feedback controller like a reflex is combined with a feedforward approach like a CPG in biological systems (Guertin, 2013). However, as shown in the fall experiments, this combination comes at the cost of decreasing the stability. In both the simulation and hardware, the adaptive CPG was overall slower to return the biomimetic leg to its limit cycle motion than the reflex. For simulated small perturbations (2–12 cm height), even the non-adaptive CPG (nF-CPG) showed a tendency to return the system faster to the limit cycle motion than the adaptive CPG. This is due to the fact that the nF-CPG was initially tuned to support the unperturbed system dynamics of the biomimetic leg. Thus, it can recover well from small disturbances, while larger perturbations might move outside the basin of attraction, making stabilizing the system eventually more challenging. In contrast, the adaptive CPG can also recover from larger disturbances, but is slower than the reflex due to the constant signal modulation.

The hardware experiments point out a further important aspect. Here, the nF-CPG performed considerably worse than the two feedback-adjusted controllers over all fall heights. We believe that this is due to the fact that manually tuning a controller to perfectly match the intrinsic dynamics of a system remains very challenging. In comparison to the feedback-adapted controllers, which drove the leg motion with 2.90 Hz, the nF-CPG resulted in a hopping frequency of 3.05 Hz. This mismatch caused an inherent offset, such that the system motion deviated even further from the limit cycle after a disturbance and the nF-CPG needed longer to recover.

Overall, the stability analysis clearly stressed how crucial the feedback component is for proper entrainment between the control signal and the mechanical system, as especially in a hardware setup manual tuning is a tedious task with unreliable results. However, a well entrained system is able to withstand small disturbances without further signal adjustments, while with larger disturbances the feedback component once more gains crucial importance. Here, a pure feedforward approach with an imminent reflex response in disturbance scenarios is more beneficial than a constantly, but slowly feedback-adapted CPG.

### 4.2. Energy Efficiency

In the simulations, the reflex controller was shown to be significantly more efficient at inserting energy into the biomimetic leg than both CPGs, mainly due to the differing shape of the reflex control signal. In contrast, in the hardware setup the performance of the adaptive CPG matched the reflex capabilities as here motor dynamics led to a similar signal shape for the different control approaches. This clearly showed that, independent of the applied control strategy, not only does the frequency of the control signal need to be adapted in changing environmental conditions, but likewise the signal shape is important.

The reflex was implemented as a bang-bang controller (Lakatos et al., 2013). Thus, in the idealized simulation, the same defined amount of energy per limit cycle was inserted instantaneously into the system when the threshold was crossed. Therefore, energy was never “lost.” In contrast, the control signal commanded by the CPGs was continuous, which is more appropriate to assume for real systems, robotic and biological ones alike. With a continuous signal, energy is added over a longer period of time, here during the stance phase. During each limit cycle the motors were commanded to counteract the joint deflection while the springs simultaneously unloaded the joints. As the springs extended faster than the motor was commanded, the joint motion pulled the motors creating the generator-effect, in which large portions of motor energy were “lost” (cf. Figure 6B). Therefore, energy could not be applied as efficiently. With the feedback modulated CPG this effect was seen to be even larger as the joint deflection was adjusted to be higher with increased loads.

The influence that the shape of the control signal exerted in driving efficient motions was verified by the hardware experiments. Here, the dynamics of the real motor overlayed the ideally given signals of the controllers leading to a throughout similar shape of the control signals. Thus, the reflex and the adaptive CPG performed similarly well. The much worse performance of the unmodulated nF-CPG can be attributed to the inherent mismatch between control signal and system dynamics. With increasing load, the frequencies of the inherent system motion and the control signal deviate further and subsequently the nF-CPG cannot effectively insert energy anymore to drive the hopping motion.

Therefore, the analysis of the energy efficiency stresses the fact that not only the adaptation of the control signal frequency is crucial for efficient motions, but also the shape of the control signal. This contrasts current work on CPGs and adaptive frequency oscillators, which usually focuses only on using the feedback component for frequency entrainment to exploit resonance effects (Buchli et al., 2006; Iwasaki and Zheng, 2006; Dzeladini et al., 2014; Khoramshahi et al., 2017; Santos et al., 2017). Our work shows that this focus should be extended to also consider and adjust the temporal shape of the control signal.

## 5. Conclusion and Outlook

The presented research applied different bioinspired control strategies with varying degrees of feedback to a biomimetic robotic leg. By assessing the system's stability and energy efficiency in simulations and hardware experiments, we quantified the contributions that reflexes and CPGs have on highly-dynamic compliant movements under different environmental influences. This can help to improve future control strategies for robotics as well as generate testable hypotheses for implemented control mechanisms in biology.

Considering robotic applications, the key findings of our research extend previous knowledge about reflexes and CPGs, which suggests the need to reevaluate current methods to combine both strategies in one controller. Commonly used adaptive CPGs are usually continuously modulated by feedback to enable proper entrainment between the system and the control signal, while also offering easy ways to modulate the gait. Conversely, the stability analysis of our work showed that such a continuous signal adaptation might not be the most beneficial option, as it is too slow to react to an imminent large disturbance. Instead, the feedback component should be applied more strategically, either to newly entrain a CPG in the case of environmental and system changes or to quickly recover the system from a large disturbance. Otherwise, the modulation of the CPG signal might not be necessary. Additionally, the analysis of the energy efficiency stressed that not only the adaptation of the control signal frequency is crucial, but the shape of the control signal matters as well. This aspect is usually not regarded in current adaptive oscillator models, but would leverage the dynamics of a given mechanical system for energy efficient motions. For future work to improve robotic control approaches based on the gained insights, our present work has also provided a valuable benchmark framework against which newly developed bioinspired control strategies can be compared.

Besides their implications for robotic controllers, our key findings can likewise be used to reconsider current hypotheses about the presence and possible implementation of reflexes and CPGs in mammals. Our findings suggest that a continuous and uniform feedback modulation of CPGs, which is assumed in many CNS models, might not be the most favorable solution to combine the known advantages of reflexes and CPGs. Instead, the modulation strength of the CPG might depend on the proximity of the joints as suggested by the principle of the proximo-distal gradient (Daley et al., 2007). The principle states that proximal joints, i.e., hip and knee, are largely modulated through feedforward control, while the feedback component seems to have a stronger influence on distal joints, i.e., the ankle. Our findings support this hypothesis. The pure reflex showed to be clearly superior to recover stable limit cycle motions. Thus, the reflex component should be strong in distal joints that first encounter external disturbances. The proximal joints experience the disturbances only indirectly and in an attenuated form, hence, needing less immediate feedback. Instead, in these joints the CPG structures could be dominant, because they seem to be mainly needed for intentional motion adjustments, i.e., speed or gait changes. Controlling motion changes from the proximal joints could be more efficient since the limb inertia can be exploited to dictate the change. Indeed, robotic implementations of the proximo-distal gradient showed that it leads to more energy efficient motions (Xiong et al., 2015). In order to expand this theory and possibly further increase energy efficiency, our findings suggest to investigate how the reflexive behavior of the distal joints might affect not only the frequency, but also the temporal shape of the control signals in the proximal joints. In this way, the findings of this work help to spark new hypotheses about the implementations of reflexes and CPGs in the mammalian CNS.

## Data Availability Statement

The original contributions presented in the study are included in the article/supplementary material, further inquiries can be directed to the corresponding author.

## Author Contributions

AS, AA-S, and PS developed the structure of the paper and derived the relevant test cases. BF supported the implementation of the simulation model. TG and DS contributed to the building of the low-level control and setup of the hardware experiments. AS carried out the simulations and hardware experiments, analyzed the data and also authored the manuscript. AA-S and PS critically revised the manuscript. All authors contributed to the article and approved the submitted version.

## Funding

This research has received funding from the European Research Council (ERC) under the European Union's Horizon 2020 Research and Innovation Programme (grant agreement no. 835284) and the Specific grant agreement no. 785907 (Human Brain Project SGA2).

## Conflict of Interest

The authors declare that the research was conducted in the absence of any commercial or financial relationships that could be construed as a potential conflict of interest.

## Publisher's Note

All claims expressed in this article are solely those of the authors and do not necessarily represent those of their affiliated organizations, or those of the publisher, the editors and the reviewers. Any product that may be evaluated in this article, or claim that may be made by its manufacturer, is not guaranteed or endorsed by the publisher.

## Footnotes

^1^ https://neurorobotics.net/index.html
